# Time-Dependent Effect of Orchidectomy on Vascular Nitric Oxide and Thromboxane A_2_ Release. Functional Implications to Control Cell Proliferation through Activation of the Epidermal Growth Factor Receptor

**DOI:** 10.1371/journal.pone.0102523

**Published:** 2014-07-11

**Authors:** Marta del Campo, Ana Sagredo, Lara del Campo, Antonio Villalobo, Mercedes Ferrer

**Affiliations:** 1 Departamento de Fisiología, Facultad de Medicina, Universidad Autónoma de Madrid, Madrid, Spain; 2 Instituto de Investigaciones Sanitarias IdIPAZ, Hospital La Paz, Madrid, Spain; 3 Instituto de Investigaciones Biomédicas, Consejo Superior de Investigaciones Científicas and Universidad Autónoma de Madrid, Madrid, Spain; University of Valencia, Spain

## Abstract

This study analyzes whether the release of nitric oxide (NO) and thromboxane A_2_ (TXA_2_) depends on the time lapsed since gonadal function is lost, and their correlation with the proliferation of vascular smooth muscle cells (VSMC) mediated by the epidermal growth factor receptor (EGFR). For this purpose, aortic and mesenteric artery segments from control and 6-weeks or 5-months orchidectomized rats were used to measure NO and TXA_2_ release. The results showed that the basal and acetylcholine (ACh)-induced NO release were decreased 6 weeks post-orchidectomy both in aorta and mesenteric artery, but were recovered 5 months thereafter up to levels similar to those found in arteries from control rats. The basal and ACh-induced TXA_2_ release increased in aorta and mesenteric artery 6 weeks post-orchidectomy, and was maintained at high levels 5 months thereafter. Since we previously observed that orchidectomy, which decreased testosterone level, enlarged the muscular layer of mesenteric arteries, the effect of testosterone on VSMC proliferation was analyzed. The results showed that treatment of cultured VSMC with testosterone downregulated mitogenic signaling pathways initiated by the ligand-dependent activation of the EGFR. In contrast, the EGFR pathways were constitutively active in mesenteric arteries of long-term orchidectomized rats. Thus, the exposure of mesenteric arteries from control rats to epidermal growth factor (EGF) induced the activation of EGFR signaling pathways. However, the addition of EGF to arteries from orchidectomized rats failed to induce a further activation of these pathways. In conclusion, this study shows that the release of NO depends on the time lapsed since the gonadal function is lost, while the release of TXA_2_ is already increased after short periods post-orchidectomy. The alterations in these signaling molecules could contribute to the constitutive activation of the EGFR and its downstream signaling pathways after long period post-orchidectomy enhancing the proliferation of the vascular muscular layer.

## Introduction

The vascular tone is regulated by several mechanisms that implicate the participation of hormonal, neuronal and endothelial factors [1]. It has been established that sex hormones are able to modify the production of different vasoactive factors released from the vessel wall. Among them, nitric oxide (NO), prostanoids and reactive oxygen species play pivotal roles regulating the vascular tone through their vasoactive properties as well as regulating cell proliferation [2]–[4]. An altered production of these factors could modify the regulation of the vascular tone leading to the development of different vascular pathologies.

Clinical studies have shown a correlation between hypotestosteronemia and incidence of cardiovascular diseases [5] and mortality risk [6]. These issues, as well as different mechanisms of action by which testosterone causes vasodilation were reviewed by Jones [7], In this regard, previous studies from our group have demonstrated an increase in the production of superoxide anion [8], prostanoids, such as thromboxane A_2_ (TXA_2_) [9], [10] and prostaglandin E_2_ (PGE_2_) [11] five months post-orchidectomy. Concerning the effect of androgens on endothelial NO release, most of studies were carried out in endothelial cells culture showing an increased release [12]–[14]. However, when the effect of androgenic derivatives was studied in orchidectomized animals, the vasodilator action of NO rather than its release was analyzed, and contradictory results were often obtained [15]–[17]. Thus, the involvement [16] or lack of involvement [17] of NO in testosterone-induced relaxation has been reported. Moreover, androgen-induced relaxation has been reported to be mediated by endothelium-independent mechanisms [18]. This variety of results could depend on the tissue, the concentration, administration-time, and the molecular structure of the androgenic derivatives used.

Concerning the effects of sex hormones deprivation on vascular function, we previously demonstrated in mesenteric artery of orchidectomized rats that the increased activity of protein kinase C (PKC) positively regulated eNOS activity [19], preventing a decrease in the release of endothelial NO. In aorta from rats, we reported that the effect of ovariectomy on NO release depended on the time lapsed since the loss of the gonads [20]. This implies that different compensatory mechanisms are likely to be at work during prolonged periods of time after gonadectomy preventing vascular failure, as already suggested [21]. In view of these data, our first objective in the present study was to determine whether there were differences in the release of NO and TXA_2_ in aorta and mesenteric arteries of rats subjected to short (6 weeks) and long (5 months) periods post-orchidectomy.

We have also found in a previous study an enlargement of the media muscular layer of mesenteric arteries from orchidectomized rats [8], suggesting that testosterone deprivation could enhance the proliferation of VSMC. The ability of TXA_2_ [22], PGE_2_ [23], superoxide anion [24] and PKC [25] to induce epidermal growth factor receptor (EGFR) transactivation have been reported, suggesting that this receptor could be implicated in the hyperproliferation of the mesenteric muscular layer from rats after 5-months post-orchidectomy. The EGFR is a receptor tyrosine kinase (RTK), which upon ligand-dependent dimerization leads to the activation of several intracellular signaling pathways, including the mitogen-activated protein kinase/extracellular-regulated kinases 1/2 (MAPK-ERK1/2) and the phosphatidylinositol 3-kinase (PI_3_K)/Akt pathways that control cell survival and cell growth [26]. In view of these data, our second objective was to analyze whether the loss of gonadal function for long periods enhances the ligand-dependent activation of the EGFR and its downstream signaling pathways in mesenteric arteries, and whether this can be correlated with the effect of testosterone on VSMC.

## Materials and Methods

### Animal protocols

Male Sprague-Dawley rats (6 months-old) were housed in the Animal Facility of the Universidad Autónoma de Madrid (registration number EX-021U) in accordance with directives 609/86 CEE and RD 233/88 of the *Ministerio de Agricultura, Pesca y Alimentación* of Spain. The animals were subjected to 12 hours of light/dark cycles and standard feeding with fodder and water *ad libitum*. In a first group, orchidectomy was performed 4 weeks after birth and 5 months later the animals were sacrificed. In a second group, the orchidectomy was performed 4.5 months after birth and 6 weeks later the animals were sacrificed. A control group not subjected to orchidectomy was also included. Surgery was performed under anesthesia by isoflurane inhalation. Absence of retraction reflex in the hind-legs after mechanical stimulation and regular respiratory rhythm were tested to determine the adequacy of anesthesia. For analgesia, rats were treated with 0.30 mg/Kg SC meloxicam (Metacam from Boehringer-Ingelheim) immediately after surgery and with 50 mg/Kg ibuprofen for 4 days. Systolic blood pressure was indirectly measured in animals 2–3 days before sacrifice by the tail-cuff method [21] using a Leica Digital Pressure Meter LE5000 (Barcelona, Spain). Extraction of blood samples (by cardiac puncture) for testosterone levels determination and body weight measurement were done the day of the experiment before the animals were sacrificed by CO_2_ inhalation. The aorta and the superior mesenteric artery were carefully dissected and removed, cleaned of connective tissue, cut into 4 mm long segments and placed in Krebs-Henseleit solution (KHS) at 4°C containing (in mM): NaCl 115, CaCl_2_ 2.5, KCl 4.6, KH_2_PO_4_ 1.2, MgSO_4_ 1.2, NaHCO_3_ 25, glucose 11.1, Na_2_-EDTA 0.03 (pH 7.4). The investigation conforms to the *Guide for the Care and Use of Laboratory Animals* published by the USA National Institutes of Health (NIH publication No. 85.23 revised 1985). This study was approved by the Ethical Committee of the *Universidad Autónoma de Madrid*.

### Testosterone levels

The testosterone level in the serum was determined using an enzyme immunoassay kit (Cayman Chemical; Ref. No. 582701). The assays were performed according to the manufacturer's instructions.

### Cells culture

Vascular smooth muscle cells (SV40LT-VSMC) were obtained from the American Type Culture Collection (ATCC) (Lot No. 3350860) and grown in Dulbecco's Modified Eagle's Medium (DMEM) supplemented with 10% (v/v) fetal bovine serum (FBS), 40 µg/ml gentamicine and 2 mM l-glutamine in F75 flasks at 37°C in a humidified atmosphere containing 5% CO_2_. Cells were seeded (450,000 cells/well) in P6 plates in the same medium, and maintained overnight in the absence of FBS and in the absence and presence of testosterone (10 nM) before performing the experiments.

### NO release

Endothelium-intact arterial segments from control and orchidectomized rats were subjected to a resting tension of 9.8 and 4.9 mN in aorta and mesenteric artery, respectively. After an equilibration period of 60 min in a buffer containing (in mM): NaCl 119, *N*-(2-hydroxyethyl)piperazine-*N*-2-ethane-sulfonic acid (HEPES) 20, CaCl_2_ 1.2, KCl 4.6, MgSO_4_ 1, NaHCO_3_ 5, glucose 5.5, KH_2_PO_4_ 0.4, Na_2_H_2_PO_4_ 0.15 (pH 7.4), the presence of vascular endothelium was tested by the ability of ACh (10 µM) to relax segments pre-contracted with noradrenaline (NA, 0.1 and 1 µM in aortic and mesenteric segments, respectively). Only arterial segments in which the ACh-induced relaxation was higher than 75% of the previous contraction were used. After, the arterial segments were rinsed several times and recovered the basal tone, arteries were incubated with the fluorescent probe 4,5-diaminofluorescein (DAF-2) (0.5 µM) for 45 min, as previously described [8]. Then, the medium was collected to measure the basal NO release. Once the organ bath was refilled, arteries were pre-contracted with NA for 2 min (0.1 µM and 1 µM in aorta and mesenteric segments, respectively), and then followed by the addition of cumulative ACh concentrations (0.1 nM–10 µM) applied at 1 min intervals to induce relaxation and to measure NO release. The fluorescence emitted by DAF-2 was measured in a spectrofluorimeter (LS50 Perkin Elmer instruments, FL WINLAB Software) using an excitation wavelength of 495 nm and an emission wavelength of 515 nm. When required assays were performed in the presence of the NO synthetase (NOS) inhibitor l-*N* ^G^-nitroarginine methyl ester (l-NAME), in which the ACh-induced fluorescence was abolished [27]. Blanks were collected from segment-free medium in order to subtract the background fluorescence. The amount of NO released was expressed as arbitrary units/mg tissue.

### Release of TXA_2_

The production of TXA_2_ *in vivo* is typically monitored by measuring its stable metabolite TXB_2_ using an enzyme immunoassay kit [11]. Endothelium-intact aortic and mesenteric arterial segments from control and orchidectomized rats were subjected to an equilibration period of 30 min in KHS at 37°C followed by 2 wash periods of 10 min using 0.2 ml of the same medium and collecting samples to measure the basal release. Once fresh KHS was replaced, arteries were exposed to NA for 2 min (0.1 µM and 1 µM in aorta and mesenteric segments, respectively), and then cumulative ACh concentrations (0.1 nM–10 µM) were applied at 1 min interval. The medium was collected, and stored at −80°C until use. To analyze the effect of testosterone on the release of TXA_2_, vascular smooth muscle cells (VSMC) were seeded (450,000 cells/well) in P6 plates and grown at 37°C. Thereafter, plates were maintained overnight in the same conditions but in the absence of FBS and in the absence and presence of testosterone (10 nM). Media were collected and stored at −80°C until use. The TXB_2_ assay was carried out according to the manufacturer's instructions. Results were expressed as pg TXA_2_/mg tissue or as pg TXA_2_/ml of medium for arteries or VSMC, respectively.

### Preparation of cell and tissue extracts

VSMC were seeded (450,000 cells/well) as previously mentioned in P6 plates and grown at 37°C. Plates were maintained overnight in the absence of FBS and in the absence and presence of testosterone (10 nM). Time-course stimulation (0–30 min) with EGF (10 nM) was carried out and the reaction was stopped with 10% (w/v) trichloroacetic acid. Artery segments were incubated in the absence and presence of EGF (10 nM) during 2 minutes. The reaction was stopped with liquid nitrogen and the arteries were stored at −80°C until used. Arterial segments were homogenated at 4°C in 150 µl of RIPA buffer containing 50 mM Tris-HCl (pH 8), 150 mM NaCl, 1% (w/v) deoxycholic acid, 1% (v/v) NP-40, 1% (v/v) SDS, 100 mM NaF, 1 mM Na_3_VO_4_, and a protease inhibitor cocktail (Calbiochem; Ref. No. 539134) supplemented with freshly prepared 1 mM phenylmethylsulfonyl fluoride. Samples were centrifuged at 16,000 g during 30 min at 4°C and the supernatant was collected to quantify protein concentration by the bicinchoninic acid assay using the BCA™ Protein Assay Kit (Pierce).

### Western blotting analysis

Electrophoresis Laemmli's loading buffer was added to each sample and heated 5 min at 100°C. Proteins were separated by SDS-PAGE in linear gradient (5–20%) gels, and transferred to polyvinylidene difluoride (PVDF) membranes (BioTRACE™). Membranes were stained with 0.1% (w/v) Fast Green in 50% (v/v) methanol and 10% (v/v) acetic acid to ascertain equal loading. Membrane segments were blocked with 5% (w/v) fat-free powdered milk or 5% (w/v) bovine serum albumin following the instructions of the antibodies' manufactures in 10 mM Tris-HCl (pH 7.4), 150 mM NaCl and 0.1% (v/v) Tween-20 (TBS-T), and incubated overnight with the following primary antibodies (Cell Signalling Technology) at a 1∶2000 dilution: monoclonal anti-phospho-tyrosine (4G10) and anti-phospho-ERK1/2 (Thr202/Tyr204); and polyclonal anti-Akt (pan), anti-phospho-Akt (S473) and anti-ERK1/2. Membranes segments were washed with TBS-T and incubated with the corresponding anti-IgG horseradish peroxidase-conjugated antibody (Invitrogene) at a 1∶5000 dilution following commercial recommendations. Finally, membranes were developed with the ECL™ Western Blotting detection kit (GE Health Care) exposing X-ray films for appropriate periods of time. The intensity of the bands was quantified with a computer-assisted scanning densitometer using the NIH Image 1.60 program.

### Statistical Analysis

All data are presented as the mean ± SEM. The experiments on NO and TXA_2_ release, testosterone level, body weight and blood pressure were analyzed using an unpaired Student's *t*-test (GraphPad Prism software), and the activation of the EGFR and signaling pathways in VSMC in the absence and presence of testosterone by the two-way analysis of variance (ANOVA). A *p*<0.05 was considered significant.

### Reagents

The reagents used were: NA, ACh, L-NAME and DAF-2 were obtained from Sigma-Aldrich, and human recombinant EGF was from PeproTech EC. Reagents were prepared in distilled water except NA that was dissolved in a solution of 0.9% (w/v) NaCl and 0.01% (w/v) ascorbic acid, and EGF that was dissolved in 25 mM HEPES-NaOH (pH 7.4). Reagent stock solutions were kept at −20°C and appropriate dilutions were made in KHS or HEPES-buffer on the day of the experiment.

## Results

### Effect of short and long periods post-orchidectomy on serum testosterone levels, body weight and blood pressure

Table 1 shows that the levels of testosterone in serum samples drastically decreased in rats 6 weeks post-orchidectomy. These levels were maintained low in 5-months post-orchidectomized animals. The body weight was not modified in 6-weeks post-orchidectomized rats, but in orchidectomized rats after 5 months it was slightly decreased. Orchidectomy did not significantly modify the systolic blood pressure in either group (Table 1). We have observed, however, that in old orchidectomized rats the systolic blood pressure was increased (control young, 141.5±4.1 mmHg; control aged, 149.2±3.8 mmHg; orchidectomized aged, 159.5±2.9 mmHg, *p*<0.05).

**Table 1:** Time-dependent effect of orchidectomy (Orch) on the serum testosterone level, blood pressure and body weight in male rats.

	Control (n = 7)	6 weeks-Orch (n = 6)	5 months-Orch (n = 8)
**Testosterone (pg/mL)**	2368±323	235±45*	220±52*
**Systolic blood pressure (mmHg)**	143±5.6	147.5±7.2	145±6.2
**Body weight (g)**	473±9.4	480±4.7	432±6.8+

The number of animal used are indicated.

*p<0.0001 *vs* control rats.

+ *p*<0.05 *vs* control rats.

### Effect of short and long periods post-orchidectomy on NO and TXA_2_ release in aorta and mesenteric arteries

**Figure 1:**
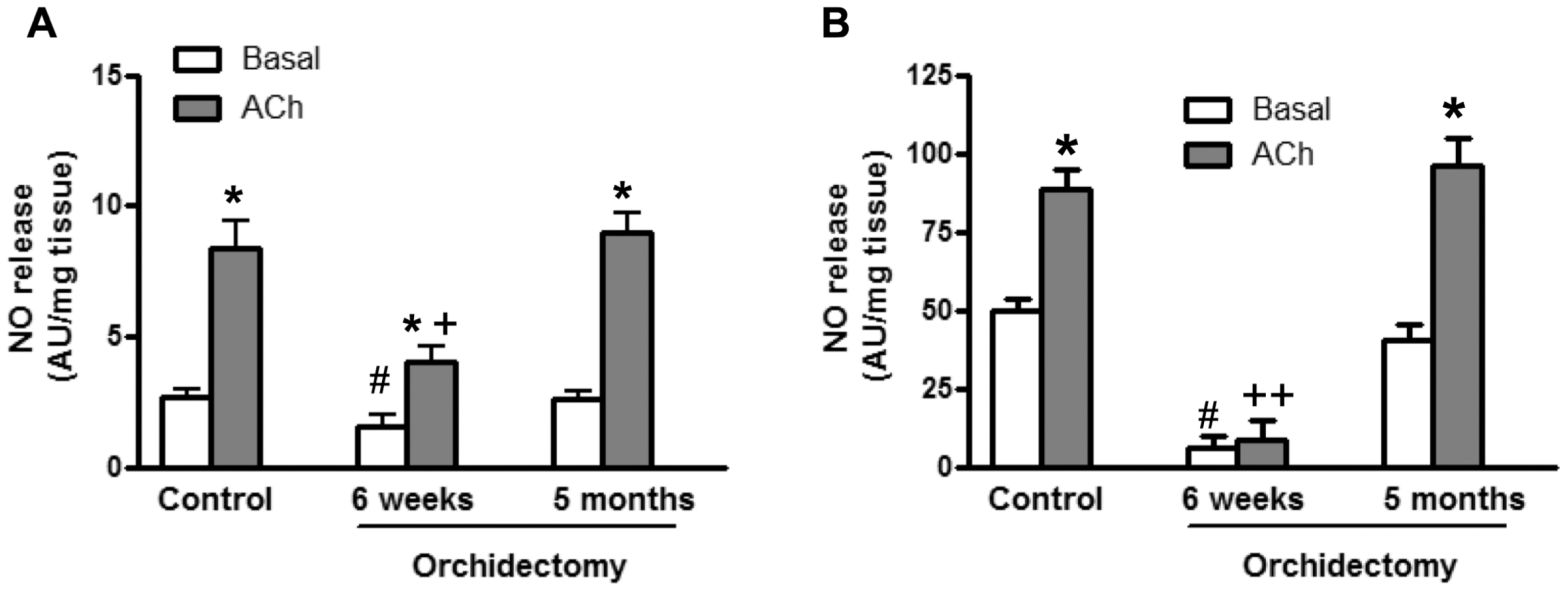
Time-dependent effect of orchidectomy on the release of NO from rat aorta and mesenteric artery. The plots present the basal- and ACh-induced NO release in aorta (A) and mesenteric (B) segments from control, 6 weeks and 5 months post-orchidectomized rats. Results (mean ± SEM) are expressed as arbitrary units (AU)/mg of tissue. * *p*<0.05, compared with its respective basal condition; # *p*<0.05 compared with basal NO release in control animals; + *p*<0.05, ^++^ *p*<0.001 compared with ACh-induced NO release in control animals. The number of animals used were: control, 6; 6 week post-orchidectomy, 4; 5 months post-orchidectomy, 7.

ACh enhanced the basal release of NO in aortic segments from control and orchidectomized rats. However, both basal and ACh-induced release of NO decreased in 6 weeks post-orchidectomized rats. These values were restored to similar levels found in aorta from control animals 5 months after orchidectomy (Fig. 1A).

Similarly, in mesenteric artery, the basal NO release was strongly decreased in 6-weeks post-orchidectomized rats, and ACh did not induce a significant further release. As observed in aortic segments, the basal and ACh-induced NO release in mesenteric artery was restored to control levels in 5-months post-orchidectomized rats (Fig. 1B).

**Figure 2:**
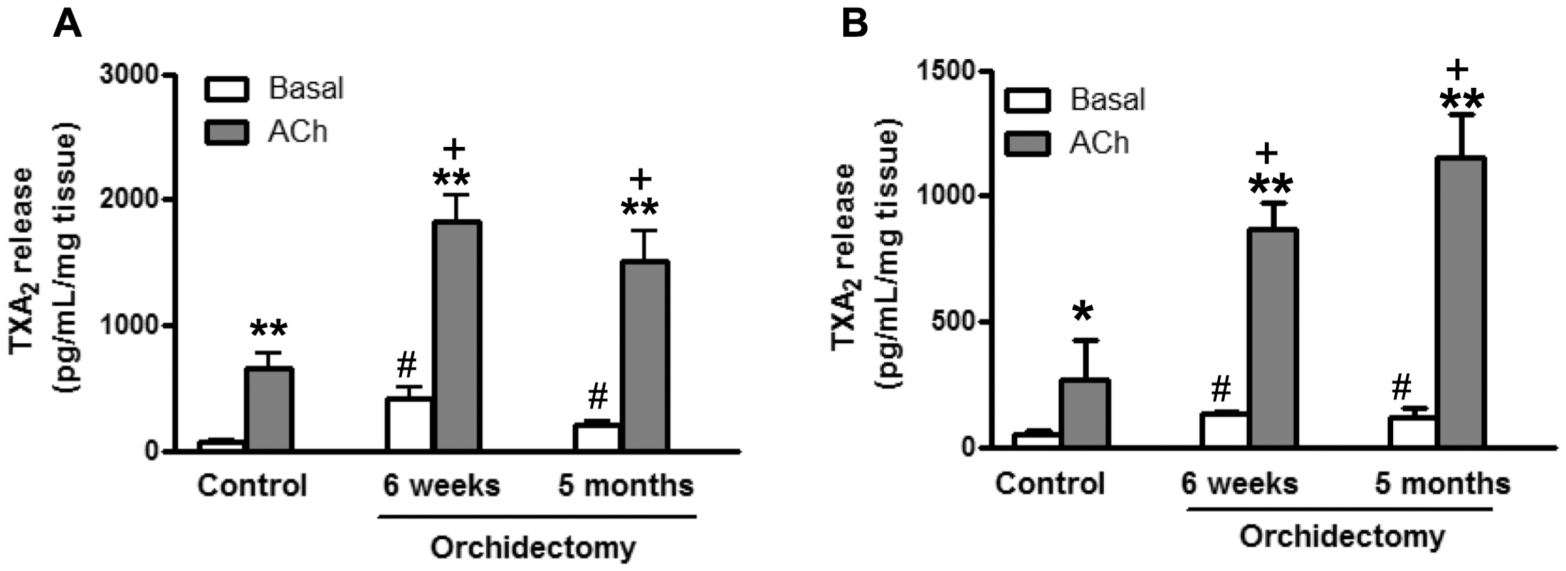
Time-dependent effect of orchidectomy on the release of TXA_2_ from rat aorta and mesenteric artery. The plots present the basal- and ACh-induced TXA_2_ release in aorta (A) and mesenteric (B) segments from control, 6 weeks and 5 months post-orchidectomized rats. Results (mean ± SEM) are expressed as pg/mL/mg tissue. Number of animals: 4–7. * *p*<0.05, ** *p*<0.001 compared with its respective basal condition; # *p*<0.01 compared with basal TXA_2_ release in control animals; + *p*<0.01 compared with ACh-induced TXA_2_ release in control animals. The number of animals used were: control, 6; 6 week post-orchidectomy, 4; 5 months post-orchidectomy, 7.

In aortic segments from control and orchidectomized rats, ACh enhanced the release of TXA_2_ [11]. Both basal and ACh-induced TXA_2_ release increased 6 weeks post-orchidectomy. This increase was not further modified in 5 months post-orchidectomized rats (Fig. 2A). Similar results to those described in aorta were found in mesenteric arteries at 6 weeks and 5 months post-orchidectomy (Fig. 2B).

The levels of TXA_2_ were also analyzed in the medium conditioned by VSMC incubated in the absence and presence of testosterone (10 nM). Our results show that after overnight incubation of VSMC with testosterone the release of TXA_2_ was decreased (control, 8.17±0.9 pg/ml; testosterone-treated, 5.4±0.3 pg/ml; n = 6, *p*<0.05).

### Effect of testosterone on the activation of EGFR signaling pathways in VSMC

**Figure 3:**
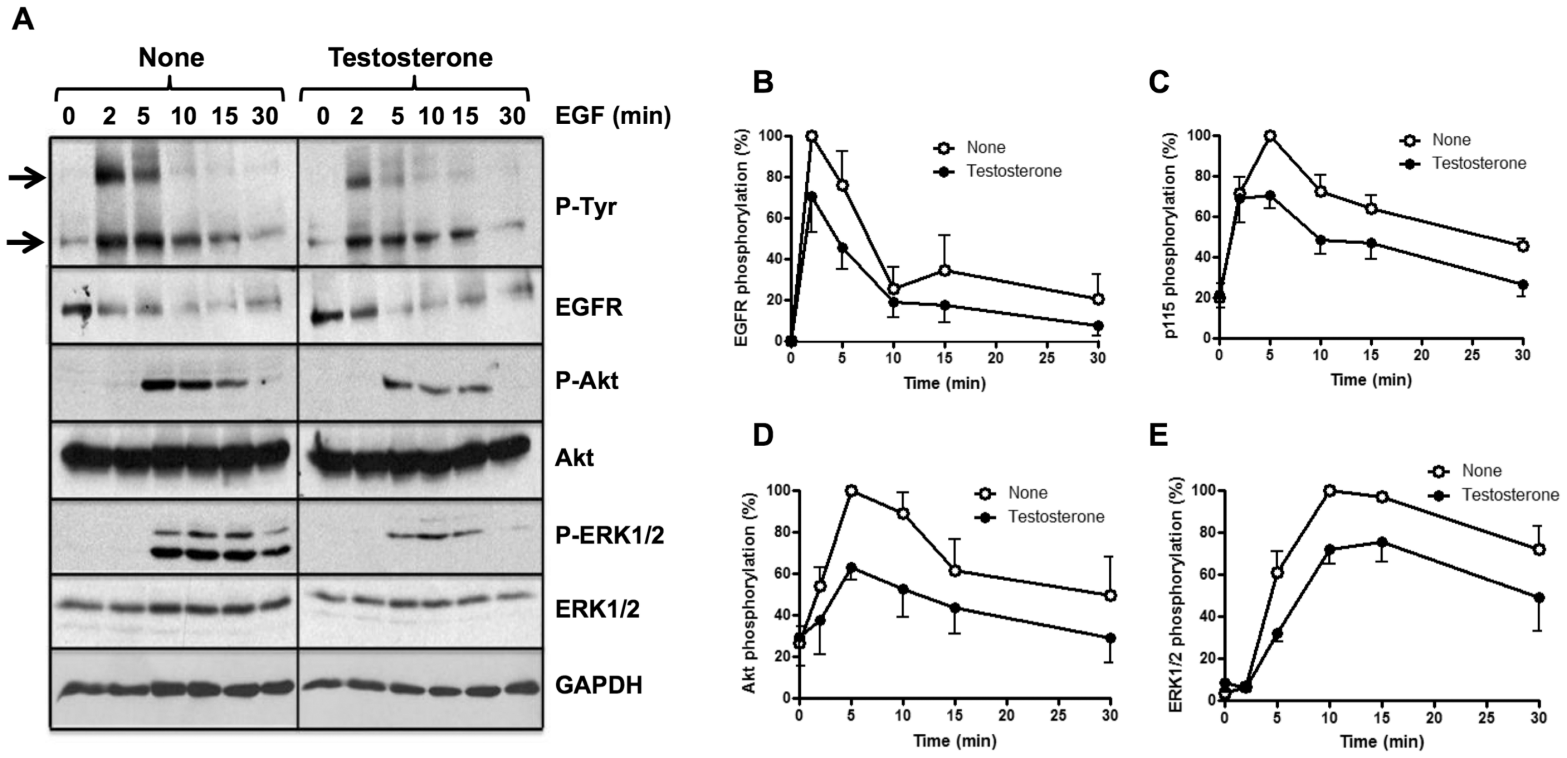
Effect of testosterone treatment on ligand-dependent activation of the EGFR and downstream signaling pathways in smooth muscle vascular cells. Serum starved SMVC were incubated with 10% (w/v) trichloroacetic acid. The samples were processed by Western blots using the indicated antibodies to determine the phosphorylation levels of the EGFR, p115, Akt, and ERK1/2, and the total levels of EGFR Akt, ERK1/2 and GAPDH as described in Materials and Methods. The total EGFR level decreased after EGF stimulation due to the expected proteolytic processing of the receptor after ligand-dependent internalization. The total Akt, ERK1/2 and GAPDH levels were used as loading controls. The intensity of the bands was measured densitometrically and the signal of the different phosphoproteins was corrected using appropriate loading controls. The photograph (A) shows typical Western blots of the proteins. The top and bottom arrows point to the phosphorylated EGFR and p115, respectively. The plots (B–E) present the mean ± SEM phosphorylation of the EGFR (n = 4) (B), p115 (n = 5) (C), Akt (n = 6) (D), and ERK1/2 (n = 6) (E) from a set of experiments similar to those shown in A.

The ligand-dependent activation of the EGFR and their downstream signaling kinases Akt and ERK1/2 were analyzed in homogenates of cultured VSMC incubated overnight in the absence and presence of 10 nM testosterone. Testosterone treatment slightly decreased the ligand-dependent phosphorylation (activation) of the EGFR after time course stimulation with 10 nM EGF (Figs. 3A and 3B). Moreover, the phosphorylation of a ≈115 kDa phospho-(Tyr)-protein (p115) (Figs. 3A and 3C), Akt (Figs. 3A and 3D) and ERK1/2 (Figs. 3A and 3E) were also significantly reduced. The EGFR was progressively dephosphorylated after addition of its ligand, and this was attributed to the combined action of phosphatases and the internalization and proteolytic processing of the receptor, as it is apparent by the progressive decrease of the total EGFR signal (Figs. 3A). Immunoprecipitation experiments showed that the ≈115 kDa phospho-(Tyr)-protein was not the catalytic subunit of PI_3_K (*data not shown*).

### Effect of long period post-orchidectomy on the activation of EGFR signaling pathways in mesenteric artery

**Figure 4:**
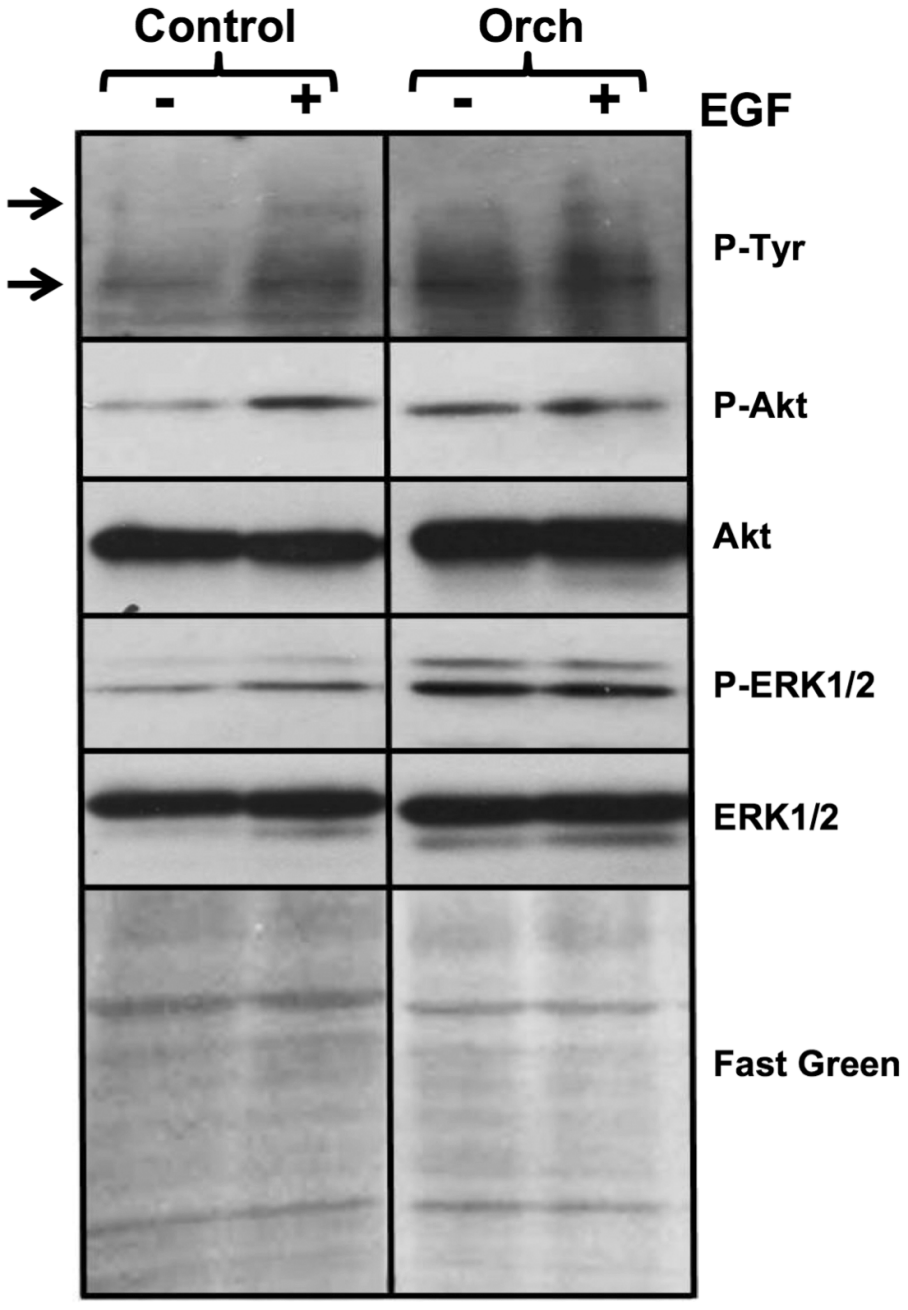
Effect of orchidectomy on the ligand-dependent activation of the EGFR and downstream signaling pathways in the mesenteric artery. Segments of the superior mesenteric artery from control and 5 months post-orchidectomized rats were incubated in the absence (−) and presence (+) of 10 nM EGF during 2 min, and the reaction was thereafter arrested freezing the samples in liquid nitrogen as described in Materials and Methods. The frozen samples were stored and thereafter homogenized and processed by Western blots using the indicated antibodies. The photograph shows the phosphorylation levels of the EGFR, p115, Akt and ERK1/2, and the total levels of Akt and ERK1/2 and segments of PVDF membranes stained with Fast Green used as additional loading control. The top and bottom arrows point to the phosphorylated EGFR and p115, respectively. The figure shows a typical experiment from control (n = 4) and orchidectomized (n = 4) rats.

We also determined the activation of the EGFR and the downstream Akt and MAPK(ERK1/2) signaling pathways in homogenates of superior mesenteric artery from control and orchidectomized rats 5 months after surgery. The stimulation of mesenteric artery from control rats with 10 nM EGF for 2 min produced the phosphorylation (activation) of a faint 170 kDa band corresponding to the EGFR. This was accompanied by a significant increased phosphorylation of the ≈115 kDa phospho-(Tyr)-protein (p115), which is likely to correspond to a substrate of the EGFR, and the phosphorylation (activation) of Akt and ERK1/2 in arteries from control rats (Fig. 4). However, in arteries from orchidectomized rats the EGFR, p115, Akt and ERK1/2 were already activated in basal conditions and did not further increase their phosphorylation upon addition of EGF (Fig. 4).

## Discussion

The pivotal role of NO and prostanoids in the regulation of the vascular tone has been established [3], [4], [28]. Likewise, the beneficial effect of androgens on the vascular function of males is widely recognized [5], [29]–[31]. The effects of androgenic derivatives on NO [12], [13] and prostanoids [32], [33] signalling pathways in different tissues have been described. Concerning NO release, both genomic [12] and non-genomic [13],[34] actions of dehydroepiandrosterone haven been reported. Testosterone has been also shown to induce NO release via activation of the PI_3_K/Akt cascade [14]. The majority of studies performed in arteries have been focused on analysing NO function instead of NO release, and discrepant results have been described. Thus, it has been reported that testosterone can increase [1], [16] and decrease [15], [17] the endothelium-dependent relaxation. However, endothelium-independent relaxation induced by testosterone and its 5-reduced metabolites has been also reported [18]. Likewise, it has been described that androgens induce vasorelaxation by activating K channels by increasing K^+^ efflux and by inhibiting calcium channels causing hyperpolarization [30]. These discrepancies could be explained depending on the tissue or animal model, the concentration, administration-time and molecular structure of the androgenic derivatives used. In addition, it is important to note that most of the published studies analyzed the effect of specific androgenic derivatives in orchidectomized animals. However, a more integrative approach could be more informative, especially taking into account that in 5 months-orchidectomized rats different signaling pathways are simultaneously working to try to maintain the vascular function [8], [19], [27], [35].

Taking into account that in aorta from female rats the basal and ACh-induced NO release was decreased 6 weeks post-ovariectomy, but recovered up to levels similar to those found in control rats 5 months thereafter [20], we analyzed whether the vessels from male rats showed a similar pattern. The results shown in the present study confirm that this is indeed the case, as both aorta and mesenteric arteries presented lower basal and ACh-induced NO release in 6 weeks post-orchidectomized rats. It is important to note that the mesenteric artery showed greater reduction in NO release than the aorta, since ACh failed to induce significant NO release in the former. This result is in agreement with that reported in mesenteric arteries of female rats, in which the involvement of endothelial NO was abolished four weeks post-ovariectomy [28]. This suggests that the effects due to the loss of gonadal function also depend on the specific vascular bed.

In addition to NO, prostanoids exert important regulatory effects on the vascular function. It has been reported that TXA_2_ is one of the most important vasoconstrictor prostanoids produced by the vascular wall that may participate in vascular dysfunction associated with cardiovascular risk factors [36]–[38]. We have already demonstrated that the release of TXA_2_ was increased 5 months post-orchidectomy in mesenteric artery [9], [10], [19], probably as consequence of increased oxidative stress observed in these experimental conditions. To ascertain whether the release of TXA_2_ also depends on the time lapsed since the gonadal function is lost, its release was also analyzed in vessels from 6 weeks post-orchidectomized rats. The results showed that after short periods of gonadal function loss the production of TXA_2_ was already increased up to similar levels of those observed in vessels from 5 months post-orchidectomized rats. This increased release of TXA_2_ over time, could be involved in the maintenance of NO release during a prolonged period of time after gonadectomy, as observed in aorta from male (*unpublished results*) and female rats [21].

Since the reduction of testosterone in the serum was already evident in 6 weeks post-orchidectomized rats and maintained 5 months post-orchidectomy, the effect of testosterone on TXA_2_ release in VSMC was analyzed. The results show that the incubation of VSMC with testosterone decreased the release of TXA_2_, in accordance with data previously reported [32]. Overall, these results indicate that testosterone is involved in the effects observed, although the participation of gonadal factors or hormones other than testosterone cannot be discarded.

It is well known that NO and TXA_2_ are able to regulate platelet aggregation and vasomotor response [38]. Moreover, it has been described that NO and TXA_2_ are important regulators of endothelial cell migration, angiogenesis and cell proliferation [39]–[42]. The participation of the EGFR in cell proliferation has been widely documented [43], [44], since its activation initiates intracellular signaling pathways with cell proliferation effects in which the kinases ERK1/2 and Akt are involved [45]. On the other hand, it has been reported that the TXA_2_ receptor transactivates the EGFR by a mechanism implicating Src-mediated phosphorylation of the receptor [25], and that NO negatively regulates the EGFR inducing the reversible S-nitrosylation of the receptor [46], [47]. Since we have found in a previous study an enlargement of the media muscular layer of mesenteric arteries from orchidectomized rats [8], we correlated the changes in NO and TXA_2_ levels with cell proliferation by analyzing the activation of EGFR-induced signaling pathway. Our results demonstrate that testosterone downregulates mitogenic signaling pathways initiated by the ligand-dependent activation of the EGFR in SMVC. Conversely, a decrease in testosterone after orchidectomy results in the basal activation of these EGFR pathways. The exposure of mesenteric arteries from control rats to EGF for 2 min induced the activation of the EGFR signaling pathways. However, in arteries from orchidectomized rats the addition of EGF did not induce a further increase in these signaling pathways. Overall, these results suggest that a decrease in the level of circulating testosterone may lead to increased proliferation of cells in the vascular wall, as previously observed in the muscular layer [8]. Likewise, the results obtained could account for the inhibitory effect of testosterone on vascular remodeling described in resistance mesenteric arteries [48]. Although the exact mechanism by which decreased level of testosterone upregulates EGFR signaling was not studied in the present work, the overproduction of TXA_2_, PGE_2_ and superoxide anion in arteries from orchidectomized rats could be involved, since transactivation of the EGFR by these signaling molecules has been described [25], [38], [49], [50], initiating the proliferation signal via the MAPK and Akt pathways. In this context, it has been reported that hypertension increased the transactivation of RTKs induced by proinflammatory mediators [51]. In the present study we reported the activation of the EGFR in mesenteric arteries after long period post-orchidectomy, and the animals did not develop hypertension, probably due to the existence of compensatory mechanisms. We believe that maintaining gonadal function is essential to prevent the development of hypertension, as only old orchidectomized animals develop hypertension. Taken together, these results could contribute to the understanding of the signaling pathways implicated in pathophysiological situations in which gonadal function is impaired. (i.e.: aging, hypogonadism, and pharmacological treatment of prostate cancer).

In summary, this study shows that the release of NO depends on the time lapsed since the gonadal function is lost, while the release of TXA_2_ is already increased after short period post-orchidectomy, and that this correlates with the possible consequences of the modifications along the time of these two crucial factors in regulating the vascular tone and the proliferation of vascular cells. In addition, this study describes for the first time the increased activation of the EGFR and its downstream signaling pathways in rat mesenteric arteries after long period post-orchidectomy, that maintained for prolonged periods of time could contribute to the development of hypertension.
